# The effectiveness of mindfulness-based stress reduction for school teachers: a cluster-randomized controlled trial

**DOI:** 10.1093/eurpub/ckab223

**Published:** 2022-02-10

**Authors:** Emilie H Bonde, Lone O Fjorback, Morten Frydenberg, Lise Juul

**Affiliations:** 1 Department of Clinical Medicine, Danish Center for Mindfulness, Aarhus University, Aarhus, Denmark; 2 MFStat, Aarhus, Denmark

## Abstract

**Background:**

Teaching has been found to be one of the most stressful occupations. Hence, current interest in reducing stress and enhancing the well-being of teachers is strong. Mindfulness-based stress reduction (MBSR) is documented to be effective in reducing stress and increasing well-being. This study investigated the effectiveness of delivering MBSR to lower secondary school teachers as a part of a teacher-training programme.

**Methods:**

This study was a nested trial within the parallel cluster-randomized controlled trial, Stress-free Everyday LiFe for Children and Adolescents REsearch (SELFCARE). Schools were recruited from all five geographical regions in Denmark between May 2018 and May 2019. One to three teachers from each school were allowed to participate. At baseline, 110 schools, representing 191 lower secondary school teachers, were cluster-randomized to intervention or a wait-list control group. The intervention group received MBSR during 2019 and the wait-list control group during 2020. Data were collected at baseline and after 3  and 6 months. The primary outcome was measured by Cohen’s Perceived Stress Scale (PSS). Data were analyzed using a mixed-effect linear regression model and bootstrapped for cluster effects.

**Results:**

At 3 months, the intervention group statistically significantly reduced their PSS score 1.7 [95% confidence interval (CI) 0.04–3.3] points more than did the wait-list control group. At 6 months, the intervention group had statistically significantly reduced their mean PSS score 2.1 (95% CI: 0.5–3.8) points more than the wait-list control group.

**Conclusion:**

It is possible to reduce perceived stress among lower secondary school teachers by delivering MBSR as part of a teacher-training programme.

## Introduction

The mental health of the European population has been eroding during the past two decades.^1^ Perceived stress is a contributor to this development. Stress is associated with depression, type-2 diabetes, cardiovascular diseases and premature mortality.^2–4^ Stress is defined as the ongoing activation of the body’s stress response without physical or mental restitution.^5^

In general, teachers report experiencing high levels of stress.^6^ Teaching has been found to be among the most stressful occupations measured on psychological well-being, physical health and job satisfaction.^7^

In Denmark, stress among school teachers is a growing problem.^8^ In 2018, every fourth Danish school teacher reported experiencing symptoms of stress.^8^ Since 2012, the proportion of school teachers experiencing symptoms of work-related stress during the past month has risen by 25.6%, measured by perceived difficulties at work and trust in one’s own ability to overcome difficulties at work.^8^ Hence, there is a call for investigating stress-reducing interventions for school teachers.

The programme *mindfulness-based stress reduction* (MBSR) supports participants build resources that help them be in the present moment and cope with stress and strains of life. Mindfulness can be defined as ‘… the awareness arising through paying attention on purpose in the present moment, non-judgmentally, in the service of self-understanding, wisdom, and compassion’.^9^ MBSR is a curriculum-based, 8-week programme delivered in a group format by a trained MBSR teacher.^10^^,^^11^

MBSR has previously been evaluated in various settings and with different study populations.^12^ Previous research has found MBSR to assist adults cope with stress and challenges of life. In effect, the authors found MBSR moderately effective in improving the mental health of adults across different target groups.^12^ Furthermore, research shows that compared to non-active control groups, mindfulness-based interventions (MBIs) are effective in reducing depression, anxiety and distress and improve well-being in non-clinical settings. However, due to heterogeneity between studies, the effects of MBIs cannot be generalized across every setting.^13^ Moreover, compared to active control groups, the authors found no superiority of MBIs.^13^

Research on mindfulness for educators has demonstrated favourable effects on mental health measures of stress, anxiety, depression, burnout and strain, and for mindfulness, emotional regulation and job performance.^14^^,^^15^ An evidence map of MBIs for workplace well-being shows mixed effects of mindfulness for educators.^16^ Hence, there is a need for further research on the effects of mindfulness for this profession.

Rose et al. have proposed two strategies to prevent illness in the population: the high-risk strategy and the population-based strategy.^17^ The aim of a population-based strategy, e.g. by providing universal interventions, is to improve the health of a wide part of the population. Using this approach, health-promoting initiatives are offered to populations with the largest parts of the populations experiencing moderate risks of adverse health, e.g. moderate stress level.^17^ This approach might yield smaller effect sizes than the high-risk strategy where interventions are offered to selected high-risk groups with more room for improvement. However, these smaller effect sizes may have a higher impact at the society level.^17^

In 2017, the Danish Parliament granted the Danish Center for Mindfulness (DCM) funding to educate school teachers in teaching mindfulness to lower secondary school children. As part of their education, the teachers received an 8-week MBSR course. The purpose of the present study was to evaluate the effectiveness of delivering MBSR to Danish lower secondary school teachers participating in a teacher training-programme compared to usual practice; measured on their perceived stress level and mental health 6 months after enrolment.

## Methods

### Design

A two-arm parallel cluster-randomized controlled trial was conducted using schools as clusters. This study was a nested trial within the research project *Stress-free Everyday LiFe for Children and Adolescents REsearch (SELFCARE)*. The project was approved by the Danish Data Protection Agency (2016-051-000001/1145). The nested trial was registered at ClinicalTrials.gov (NCT03886363) in March 2019.^18^

### Setting

The trial was conducted across all five geographical regions in Denmark. At present, there are 1326 municipal schools (71%) and 538 (29%) private schools in Denmark.^19^ This study, being a nested trial, was conducted within the setting of the main trial described in the protocol.^18^

### Participants

School teachers from private and municipal schools in all five geographical regions of Denmark were included. To be included, schools were required to have ≥100 pupils. Furthermore, the headmaster/mistress had to give consent for teachers to participate in the trial and to allow the teachers to spend working hours participating. Participants were recruited between May 2018 and May 2019 through advertisements on the DCM webpage, social media posts, invitational letters to schools in selected regions and local information meetings. In total, 110 schools enrolled. The individual schools chose which teachers to enrol. A maximum of three teachers was allowed to enrol from each school.

### Procedure and randomization

**Figure 1:**
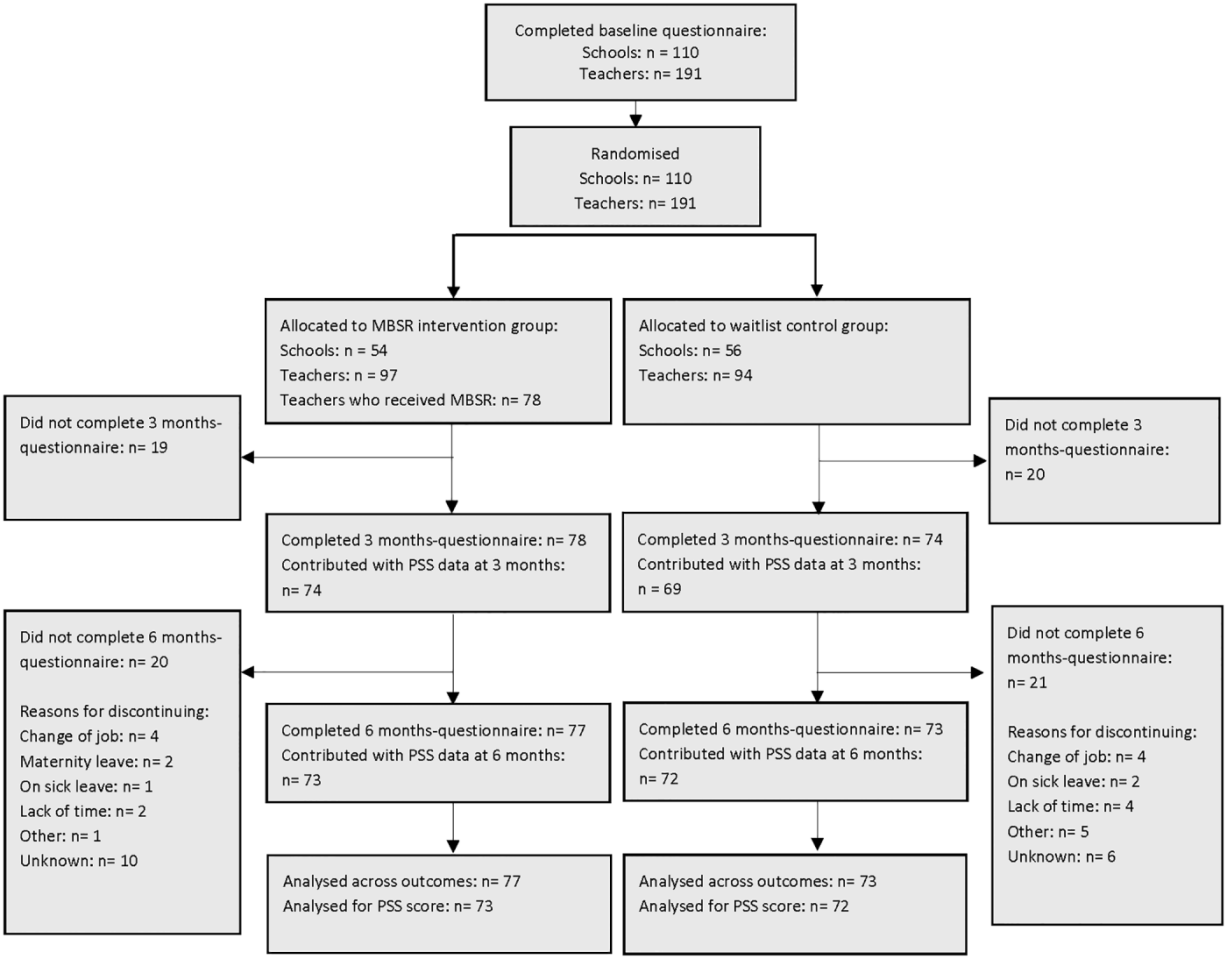
Flow chart of participants

All participants were informed about the trial and use of data. Teachers provided consent by completing the baseline questionnaire, this being standard protocol in Denmark when conducting non-biological research.^20^ Data were collected using Research Electronic Data Capture (REDCap). REDCap is a secure online platform for managing data collection for research.^21^ REDCap was used to build and distribute questionnaires via e-mail. Schools were randomized to begin teacher-training in 2019 or 2020. Block-randomization was performed in five blocks corresponding to the geographical regions. For each region, the third author received a list with anonymized school ids. The randomization was stratified by school size (more or less than 500 pupils), school type (private or municipal) and number of teachers included in the trial (1 or 2–3). Finally, the anonymous school ids were linked to the schools’ actual identity.^18^ Randomization was conducted between February 2019 and September 2019. The allocation ratio was 1:1. The data collectors were not blinded to group assignment, as they were able to identify school affiliations in data collected using REDCap. In total, 191 school teachers contributed with baseline data and were included in this trial (figure 1).

### Intervention

#### Intervention group

The intervention group began teacher training in 2019. As part of the teacher-training programme, the participants received an 8-week MBSR course in a group of 8 to 28 school teachers from the same geographical region. MBSR is a curriculum-based course consisting of 8 weekly 2.5-hour sessions and a 7-hour silent retreat day. The participants are invited to practice mindfulness for 60 minutes during the day 6 days a week. The aim of the MBSR course is to support the participants in developing their own mindfulness practice.^10^^,^^11^ The course must be delivered by a trained MBSR teacher. The MBSR teachers delivering the courses in this trial were employed by the DCM but were not part of the research group. The second author supervised the MBSR teachers. The remaining elements of the teacher-training programme were delivered after 6 months of follow-up and are outside the scope of the present study.^18^

#### Wait-list control group

The teachers representing schools randomized to the control group were put on the waiting list to receive the teacher-training programme including an MBSR course in 2020.

### Outcomes and instruments

The primary outcome was measured by Cohen’s Perceived Stress Scale (PSS)^22^ 6 months from baseline. Primary outcome measure at 6 months of follow-up was chosen to allow for the longest follow-up period strictly relating to MBSR before introducing the remaining teacher-training elements. Secondary outcomes were well-being and symptoms of depression and anxiety as well as proposed mediators such as dispositional mindfulness. Data were collected at baseline, 3 and 6 months from baseline.

#### Perceived Stress Scale

The 10-item version of PSS measures subjective stress.^22^ The items investigate how often during the past month respondents have experienced life as unpredictable, uncontrollable and overloaded. Items are scored on a five-point Likert scale (sum scores 0–40) with higher scores indicating higher levels of perceived stress. The scale has shown to be valid and reliable.^23–25^

#### The Hopkins Symptom Checklist-5

The five-item version of the Symptom Checklist is a self-report measure of symptoms of depression and anxiety.^26^ Items are scored on a four-point scale. An average score is calculated with higher scores indicating higher levels of self-reported symptoms of depression and anxiety. This shortened version of the original 25-item SCL correlates at *r* = 92. The alpha reliability for the SCL-5 is estimated to be 0.85.^27^

#### The WHO-5 Wellbeing Scale

The World Health Organization-Five Well-Being Index (WHO-5) is a five-item self-report measure of well-being.^28^ The items investigate how often during the past 2 weeks the respondents have experienced specific feelings. Items are scored on a five-point scale. A total score is calculated by adding and multiplying sub scores, providing a score range from 0 to 100. Higher scores indicate higher levels of well-being. WHO-5 is considered to be a valid measure of individuals’ well-being.^28^

#### Brief Resilience Scale

The Brief Resilience Scale (BRS) is a six-item measure of the respondents’ resilience. Items are scored on a five-point scale, and an average score is calculated. Higher scores indicate greater resilience.^29^ Cut-points of low, normal and high resilience have been proposed; low: 1.00–2.99, normal: 3.00–4.30 and high: 4.31–5.00. BRS has been suggested to be one of the most valid instruments to measure resilience among adults.^30^

#### The Five Facet Mindfulness Questionnaire

The Five Facet Mindfulness Questionnaire-15 is a 15-item self-report measure of a respondent’s dispositional mindfulness^31^ and includes five facets of mindfulness: Observing, Describing, Acting with awareness, Non-judgement and Non-reactivity. Items are scored on a five-point scale. A total score is calculated by summing the scores of each sub-scale and then summing the sub-scores into one total score with higher scores indicating higher dispositional mindfulness. Gu et al. have suggested that the sub-score for the facet ‘observing’ be omitted when calculating the total score.^32^ When omitting this sub-score, the range of the total score is 12 to 60. The FFMQ-15 is a shortened version of the original FFMQ-39 and has been found valid and reliable.^31^

#### The Amsterdam Resting State Questionnaire

The Amsterdam Resting State Questionnaire (ARSQ) is a self-report measure of thoughts and feelings in rest, containing 21 statements, across 7 dimensions: Discontinuity of Mind, Theory of Mind, Self, Planning, Sleepiness, Comfort and Somatic Awareness. Each item is scored on a five-point Likert scale.^33^ Scores for each of the seven dimensions are calculated as sum scores. Each dimension has a score range from 3 to 15.

### Statistical analysis

Before the trial commenced, a power calculation was performed. Based on previous research, the expected mean effect on PSS score was −2.5 score points (SD 5.8).^31^ To detect this effect with a power of 80%, the trial had to include 86 teachers in each group; 172 in total.

Data from the three time points were analyzed in a mixed-effect linear regression model with systematic effect of randomization, time, interaction between randomization and time, sex, age (continuous variable), school type (municipal or private), school size (1–499 or 500+ pupils) and geographical region. As one MBSR course was delivered in each region, the ‘region’ variable also represents clusters of MBSR courses. We assumed random effect of school and teacher. Standard errors and confidence intervals (CIs) were based on bootstrapping, resampling teachers and giving each resampled teacher a new unique id.

The data were analyzed according to the intention-to-treat principle (ITT), i.e. all available data from participants were analyzed according to the randomization group the participants were originally assigned, regardless of what intervention they received (e.g. whether the participants in the intervention group completed the MBSR course or not).

Cohen’s d was estimated for all outcomes. The following cut-points for interpretation of the Cohen’s d were used; small effects: 0.2, medium effects: 0.5 and large effects: 0.8.^34^ Regarding loss to follow-up, (i) analyses of loss to 6 months follow-up were performed using *t* tests and χ^2^ tests (Supplementary tables S1–S3). Two-sided *P* values were applied and statistical significance was set at *P* = 0.05 and (ii) sensitivity analyses were conducted. In these analyses, model-based predictions were performed by adding or subtracting 0.2 SD in either the intervention arm or the control arm (Supplementary tables S4 and S5).^18^ Analyses were performed using Stata 16.1 software.

## Results

### Baseline characteristics

The intervention group consisted of 97 teachers and the wait-list control group of 94 teachers. Participants were mainly female (91.6%) with an average age of 45.2 (SD 8.4) years at baseline. The majority of participants represented municipal schools (67.5%) (table 1). The study population’s mean PSS score at baseline was 15.8 (SD 5.7). Generally, the two groups were comparable at baseline across demography and self-reported mental health scores (table 1).

**Table 1:** Characteristics of school teachers at baseline

	MBSR intervention (*n* = 97)		Wait-list control (*n* = 94)		Total (*n* = 191)	
	Included	Missing, *n* (%)	Included	Missing, *n* (%)	Included	Missing, *n* (%)
Characteristic						
Sex, *n* (%)						
Men	10 (10.3)	0 (0)	6 (6.4)	0 (0)	16 (8.4)	0 (0)
Women	87 (89.7)	0 (0)	88 (93.6)	0 (0)	175 (91.6)	0 (0)
Age, mean (SD), year	46.2 (8.7)	0 (0)	44.2 (8.1)	0 (0)	45.2 (8.4)	0 (0)
Geographical region, *n* (%)						
Central Denmark Region	26 (26.8)	0 (0)	24 (25.5)	0 (0)	50 (26.2)	0 (0)
The Capital Region of Denmark	29 (29.9)	0 (0)	27 (28.7)	0 (0)	56 (29.3)	0 (0)
Region Zealand	14 (14.4)	0 (0)	14 (14.9)	0 (0)	28 (14.7)	0 (0)
The Region of Southern Denmark	21 (21.7)	0 (0)	21 (22.4)	0 (0)	42 (22.0)	0 (0)
The North Denmark Region	7 (7.2)	0 (0)	8 (8.5)	0 (0)	15 (7.8)	0 (0)
School type (%)						
Private	33 (34.0)	0 (0)	29 (30.9)	0 (0)	62 (32.5)	0 (0)
Municipal	64 (66.0)	0 (0)	65 (69.1)	0 (0)	129 (67.5)	0 (0)
School size (%)						
≤499 pupils	50 (51.6)	0 (0)	50 (53.2)	0 (0)	100 (52.4)	0 (0)
≥500 pupils	47 (48.4)	0 (0)	44 (46.8)	0 (0)	91 (47.6)	0 (0)
Self-reported mental health [mean (SD)]						
PSS	15.4 (5.4)	3 (3.1)	16.2 (6.0)	2 (2.1)	15.8 (5.7)	5 (2.6)
SCL-5	1.9 (0.5)	1 (1.0)	1.9 (0.6)	1 (1.1)	1.9 (0.5)	2 (1.0)
WHO-5	59.7 (16.9)	1 (1.0)	58.6 (17.1)	2 (2.1)	59.1 (17.0)	3 (1.6)
BRS	4.3 (0.9)	2 (2.1)	4.3 (0.8)	2 (2.1)	4.3 (0.9)	4 (2.1)
FFMQ-15	41.8 (5.5)	3 (3.1)	42.0 (5.7)	4 (4.3)	41.9 (5.6)	7 (3.7)
ARSQ						
Discontinuity of Mind	9.0 (2.6)	1 (1.0)	9.0 (2.8)	4 (4.3)	9.0 (2.7)	5 (2.6)
Theory of Mind	8.6 (2.8)	3 (3.1)	9.2 (2.7)	3 (3.2)	8.9 (2.7)	6 (3.1)
Self	9.2 (2.3)	2 (2.1)	9.6 (2.0)	2 (2.1)	9.4 (2.2)	4 (2.1)
Planning	9.0 (2.9)	2 (2.1)	9.7 (2.9)	3 (3.2)	9.4 (2.9)	5 (2.6)
Sleepiness	6.6 (2.6)	4 (4.1)	6.4 (2.3)	3 (3.2)	6.5 (2.5)	7 (3.7)
Comfort	10.7 (1.9)	3 (3.1)	10.6 (2.0)	2 (2.1)	10.7 (2.0)	5 (2.6)
Somatic awareness	10.4 (2.2)	2 (2.1)	10.6 (2.3)	2 (2.1)	10.5 (2.2)	4 (2.1)

ARSQ, Amsterdam Resting-State Questionnaire; BRS, Brief Resilience Scale; FFMQ, Five Facet Mindfulness Questionnaire; MBSR, Mindfulness-based Stress Reduction; *n*, number; PSS, Cohen’s Perceived Stress Scale; SCL-5, The Hopkins Symptom Checklist 5; SD, standard deviation; WHO-5, WHO-5 Well-being Scale.

### Attendance

Of the 97 school teachers allocated to the intervention group, 78 (82%) participated in an MBSR course (figure 1). None of the 78 participants attended fewer than five MBSR sessions with an average attendance of 7.6 sessions out of 9.

### Effectiveness

Table 2 shows the effect of MBSR compared to usual practice after 3 and 6 months among lower secondary school teachers participating in MBSR as part of a teacher-training programme. The intervention group reduced their mean PSS score by 1.7 (95% CI: 0.04–3.3) points more after 3 months than the wait-list control group. Furthermore, the between-group difference increased from 3 to 6 months. As such, the intervention group reduced their mean PSS score by 2.1 (95% CI: 0.5–3.8) points more than the wait-list control group after 6 months. Hence, the effect of MBSR on PSS was statistically significant. Our study did not show statistically significant effect of MBSR on other mental health outcomes. However, tendencies pointed towards favouring the MBSR group. The intervention group reduced symptoms of depression and anxiety at 3 months by 0.2 (95% CI: −0.01 to 0.3) point more than the wait-list control group. At 6 months, the difference in reduction was 0.1 (95% CI: −0.04 to 0.2) point. Moreover, tendencies indicated that the well-being of the school teachers increased more in the intervention group than in the wait-list control group at both 3 months, WHO-5: 4.9 (95% CI: −0.1 to 9.9) points and at 6 months: 3.6 (95% CI: −1.7 to 9.0) points.

**Table 2:** Effectiveness of MBSR for school teachers on perceived stress, symptoms of anxiety and depression and wellbeing at 3- and at 6-month follow-up (mixed model analysis)

	MBSR intervention			Wait-list control					
Measure	Teachers (*n*)	Score, mean (SD)	Within-group change from baseline, mean (95% CI)^a^	Teachers (*n*)	Score, mean (SD)	Within-group change from baseline, mean (95% CI)^a^	Between-group difference, mean (95% CI)^a^	*P* value	Cohen’s d
PSS^b^									
Baseline	94	15.4 (5.4)	NA	92	16.2 (6.0)	NA	NA	NA	NA
3 months	74	13.1 (5.3)	−2.2 (−3.6 to −0.8)	69	15.8 (6.3)	−0.5 (−1.6 to 0.6)	−1.7 (−3.3 to −0.04)	0.04	0.30
6 months	73	12.9 (5.0)	−2.3 (−3.5 to −1.1)	72	16.1 (5.9)	−0.2 (−1.2 to 0.8)	−2.1 (−3.8 to −0.5)	0.01	0.37
SCL−5^c^									
Baseline	96	1.9 (0.5)	NA	93	1.9 (0.6)	NA	NA	NA	NA
3 months	76	1.7 (0.5)	−0.2 (−0.3 to −0.06)	69	1.9 (0.6)	−0.02 (−0.1 to 0.09)	−0.2 (−0.3 to 0.01)	0.07	0.30
6 months	74	1.7 (0.4)	−0.1 (−0.2 to −0.03)	72	1.9 (0.6)	−0.04 (−0.1 to 0.07)	−0.1 (−0.2 to 0.04)	0.16	0.19
WHO-5^d^									
Baseline	96	59.7 (16.9)	NA	92	58.6 (17.1)	NA	NA	NA	NA
3 months	74	64.1 (15.2)	3.9 (−0.6 to 7.2)	70	57.6 (18.2)	−1.0 (−5.0 to 3.0)	4.9 (−0.1 to 9.9)	0.05	0.29
6 months	74	62.6 (14.6)	2.3 (−1.5 to 6.1)	73	57.1 (17.1)	−1.3 (−4.7 to 2.0)	3.6 (−1.7 to 9.0)	0.18	0.21

a Adjusted for systematic effect: randomization, sex, age, region, school type, school size and school cluster effect.

b Measure of perceived stress with higher values indicating higher levels of perceived stress.

c Measure of self-reported symptoms of depression and anxiety with higher values indicating higher levels of self-reported symptoms of depression and anxiety.

d Measure of general wellbeing with higher values indicating higher level of wellbeing.

CI, confidence interval; MBSR, Mindfulness-based Stress Reduction; *n*, number; NA, not applicable; PSS, Cohen’s Perceived Stress Scale; SCL-5, The Hopkins Symptoms Checklist-5; SD, standard deviation; WHO-5, The WHO-5 Well-being Scale.

Table 3 shows the estimated effect in proposed mediators. Measured by BRS, there was no statistically significant effect on resilience after 3 months: 0.1 (95% CI: −0.1 to 0.4) point and after 6 months: −0.003 (95% CI: −0.2 to 0.2) point. Measured by FFMQ-15, the intervention group reported statistically significantly higher mean dispositional mindfulness than the wait-list control group after 3 months: 1.6 (95% CI: 0.2–2.9) points. However, at 6 months, this difference was no longer statistically significant: 0.9 (95% CI: −0.2 to 2.0) point. Furthermore, the intervention group reported statistically significantly more comfort [0.9 (95% CI: 0.05–1.7)] and statistically significantly more bodily awareness [1.0 (95% CI: 0.1–2.0)] in rest at 6 months than did the wait-list control group. The intervention group also experienced statistically significantly less discontinuity of mind [−0.9 (95% CI: −1.8 to −0.01)] during rest at 6 months than did the wait-list control group.

**Table 3:** Effectiveness of MBSR for school teachers on resilience, dispositional mindfulness and thoughts and feelings in rest at 3- and 6-month follow-up (mixed model analysis)

	MBSR intervention			Wait-list control					
Measure	Teachers (n)	Score, mean (SD)	Within-group change from baseline, mean (95% CI)^a^	Teachers (n)	Score, mean (SD)	Within-group change from baseline, mean (95% CI)^a^	Between-group difference, mean (95% CI)^a^	*P* value	Cohen’s d
BRS^b^									
Baseline	95	4.3 (0.9)	NA	92	4.3 (0.8)	NA	NA	NA	NA
3 months	74	4.5 (0.8)	0.2 (0.02 to 0.4)	68	4.3 (0.9)	0.05 (−0.1 to 0.2)	0.1 (−0.1 to 0.4)	0.22	0.16
6 months	73	4.4 (0.9)	0.05 (−0.1 to 0.2)	71	4.3 (0.9)	0.05 (−0.1 to 0.2)	−0.003 (−0.2 to 0.2)	0.98	0.004
FFMQ−15^c^									
Baseline	94	41.8 (5.5)	NA	90	42.0 (5.7)	NA	NA	NA	NA
3 months	71	43.6 (5.5)	1.4 (0.4 to 2.4)	68	41.7 (5.5)	−0.2 (−1.3 to 0.9)	1.6 (0.2 to 2.9)	0.02	0.28
6 months	72	43.3 (4.9)	0.9 (−0.02 to 1.8)	68	41.7 (5.0)	−0.05 (−0.9 to 0.8)	0.9 (−0.2 to 2.0)	0.09	0.17
ARSQ^d^									
Discontinuity of mind									
Baseline	96	9.0 (2.6)	NA	90	9.0 (2.8)	NA	NA	NA	NA
3 months	76	7.6 (2.6)	−1.4 (−1.9 to −0.9)	69	9.0 (2.8)	−0.01 (−0.6 to 0.5)	−1.4 (−2.2 to −0.6)	<0.01	0.52
6 months	73	8.1 (2.6)	−0.9 (−1.6 to −0.2)	73	9.1 (2.4)	0.03 (−0.6 to 0.7)	−0.9 (−1.8 to −0.01)	0.05	0.34
Theory of mind									
Baseline	94	8.6 (2.8)	NA	91	9.2 (2.7)	NA	NA	NA	NA
3 months	75	8.3 (2.7)	−0.3 (−0.9 to 0.3)	69	9.3 (2.7)	0.2 (−0.6 to 1.0)	−0.5 (−1.5 to 0.5)	0.31	0.19
6 months	73	8.9 (2.5)	0.4 (−0.2 to 1.0)	72	9.8 (2.6)	0.7 (0.2 to 1.1)	−0.3 (−1.1 to 0.5)	0.49	0.10
Self									
Baseline	95	9.2 (2.3)	NA	92	9.6 (2.0)	NA	NA	NA	NA
3 months	74	8.9 (2.2)	−0.3 (−0.8 to 0.2)	69	9.5 (2.4)	−0.2 (−0.8 to 0.5)	−0.1 (−0.9 to 0.7)	0.79	0.05
6 months	71	9.4 (2.4)	0.2 (−0.4 to 0.8)	72	9.3 (2.2)	−0.4 (−1.1 to 0.2)	0.6 (−0.2 to 1.4)	0.14	0.28
Planning									
Baseline	95	9.0 (2.9)	NA	91	9.7 (2.9)	NA	NA	NA	NA
3 months	76	8.1 (2.8)	−0.9 (−1.7 to −0.2)	66	10.3 (2.4)	0.6 (−0.2 to 1.4)	−1.5 (−2.6 to −0.4)	0.01	0.53
6 months	72	8.6 (2.7)	−0.3 (−1.0 to 0.4)	71	9.9 (2.8)	0.1 (−0.6 to 0.9)	−0.4 (−1.4 to 0.6)	0.42	0.14
Sleepiness									
Baseline	93	6.6 (2.6)	NA	91	6.4 (2.3)	NA	NA	NA	NA
3 months	76	6.1 (2.8)	−0.3 (−1.1 to 0.4)	68	7.1 (2.5)	0.7 (0.03 to 1.4)	−1.0 (−2.0 to −0.02)	0.05	0.42
6 months	72	6.2 (2.8)	−0.2 (−0.9 to 0.6)	73	7.2 (2.4)	0.7 (0.1 to 1.3)	−0.9 (−1.9 to 0.3)	0.10	0.35
Comfort									
Baseline	94	10.7 (1.9)	NA	92	10.6 (2.0)	NA	NA	NA	NA
3 months	75	10.9 (2.1)	0.2 (−0.2 to 0.6)	70	10.4 (2.1)	−0.3 (−0.7 to 0.1)	0.5 (−0.1 to 1.1)	0.07	0.28
6 months	73	11.1 (1.9)	0.4 (−0.1 to 0.9)	73	10.2 (2.3)	−0.5 (−1.1 to 0.2)	0.9 (0.05 to 1.7)	0.04	0.44
Somatic awareness									
Baseline	95	10.4 (2.2)	NA	92	10.6 (2.3)	NA	NA	NA	NA
3 months	74	10.9 (2.3)	0.4 (−0.1 to 1.0)	68	10.2 (2.4)	−0.6 (−1.3 to 0.2)	1.0 (−0.1 to 2.0)	0.07	0.44
6 months	72	10.5 (2.4)	0.03 (−0.6 to 0.6)	72	9.7 (2.4)	−1.0 (−1.7 to −0.3)	1.0 (0.1 to 2.0)	0.03	0.46

a Adjusted for systematic effect randomization, sex, age, region, school type, school size and school cluster effect.

b Measure of resilience with higher values indicating higher levels of resilience.

c Measure of dispositional mindfulness with higher values indicating higher levels of dispositional mindfulness.

d Measure of thoughts and feelings in rest with higher values indicating more frequent experience of the seven sub-dimensions in rest.

CI, confidence interval; ARSQ, Amsterdam Resting-State Questionnaire; BRS, Brief Resilience Scale; FFMQ, Five Facet Mindfulness Questionnaire; MBSR, Mindfulness-based Stress Reduction; *n*, number; NA, not applicable; SD, standard deviation.

Most effect estimates corresponded to small or medium standardized effects sizes (tables 2 and 3). However, some results showed effect sizes below 0.2.

Loss to 6-month follow-up showed statistically significant differences in age, geographical region, sleepiness and bodily awareness (Supplementary table S1–S3). The sensitivity analyses showed statistically significant effects on PSS in all four scenarios we analyzed (Supplementary table S4). Sensitivity analyses reviled that when adding 0.2 × SD to the intervention group, effects in well-being became statistically significant at both 3 and 6 months. The same was true when subtracting 0.2 × SD in the wait-list control group (Supplementary table S4). Sensitivity analyses of symptoms of depression and anxiety showed that adding 0.2 × SD to the wait-list control group provided statistically significant effects at 3 months. As did subtracting 0.2 × SD in the intervention group. The sensitivity analyses did not change the conclusions of the remaining outcomes.

## Discussion

In this study, MBSR was investigated as a health-promoting and primary preventive intervention delivered as a part of a school teacher-training programme. The study population included lower secondary school teachers, who had an interest in teaching mindfulness in schools. Their stress level was moderate at baseline and therefore with no large room for improvement. Our findings indicate that MBSR has a small significant effect in reducing perceived stress among lower secondary school teachers participating in a teacher-training programme 6 months after enrolment. According to Rose, small effect sizes found in a study that applies a population-based preventive strategy may have great implications on the society level.^17^ It may prevent the school teachers with moderate stress levels from developing high stress levels and related consequences. Previous research has documented an association between a PSS score ≥16 and the risk of long-term sickness absence from work.^35^ No other statistically significant effects were found on mental health outcomes in this study. However, there were tendencies for the intervention group to have higher levels of well-being and lower levels of symptoms of depression and anxiety than the wait-list control group at both 3 and 6 months. Furthermore, the intervention group experienced statistically significantly less discontinuity of mind and more comfort and bodily awareness in rest after 6 months than the wait-list control group.

In line with our results, previous research has shown similar effects of the stress-reducing properties of mindfulness for educators.^15^^,^^36^^,^^37^ A systematic review of mindfulness-based interventions for teachers shows a small to moderate effect with regard to stress reduction.^15^ A non-randomized feasibility trial shows a greater effect of an MBI for teachers on perceived stress than that of the present study.^36^ However, this non-randomized trial included teachers with a mean PSS score of ≥19 in the intervention group. Hence, they constituted a high-risk group. As opposed to our study, the abovementioned studies find positive effects on symptoms of depression and anxiety and on well-being.^15^^,^^36^ The lack of effects on these mental health outcomes in our study may be due to missing follow-up data (Supplementary table S4) or a smaller room for improvement in the present study.

Besides stress-reducing effects, mindfulness has been shown to assist teachers in providing genuine care for their students and creating ‘calmer and more focused classroom environments’.^38^ This illustrates the importance for teachers acquiring these personal and professional competencies.

### Strengths and limitations

Firstly, a strength of this study is its cluster-randomized design and the use of robust statistical analysis. Using mixed-effect analysis and bootstrapping, we adjusted for the cluster effects. Secondly, the study population includes schools from all five geographical regions in Denmark and represents both private and municipal schools. Hence, this study is assumed to be representative of the effects one could expect to find upon replication in a Danish setting. Thirdly, the research area of mindfulness for teachers is characterized by heterogeneity of the MBIs being evaluated.^37^ Thus, it is a strength of the present study that we employed the MBSR programme for which there is evidence for stress-reducing effect.^12^

The study also has limitations. It was conducted as a nested trial and as part of a teacher-training programme in which additional programme elements were included after 6 months follow-up. Therefore, it was not possible to measure long-term effects of MBSR past 6 months. Still, previous research has mainly used less than 6 months of follow-up.^13^ Further, the study being a nested trial prohibited the use of an active control group, as this might affect the results of the main trial. Since we compared an MBSR-intervention group to a passive wait-list control group, we cannot conclude on any specific effects of MBSR. Moreover, it cannot be ruled out that the participating self-selected school teachers might have had a pre-existing interest in mindfulness. Therefore, they could be more motivated for participation in an MBSR course compared to an average Danish school teacher. If this is the case, results of this study may solely be representative of school teachers with an interest for mindfulness.

Loss to 6-month follow-up analyses showed differences in various parameters. To mitigate the possibility of bias, sensitivity analyses were performed. The results of these did not change the main conclusions of the study. Furthermore, only self-reported measures were utilized. Since this study investigated the *perceived* stress and well-being of participants, self-reported data were deemed appropriate. However, the study could have benefitted from supplementary knowledge of whether the intervention group experienced fewer sick days than the control group.

## Conclusions

This study shows that it is possible to reduce the perceived stress level of lower secondary school teachers by offering MBSR as part of a teacher-training programme educating lower secondary school teachers to teach mindfulness in schools. The difference in perceived stress level between the intervention and control groups continued to increase from 3 to 6 months. Teachers in the intervention group reported moderate a mean perceived stress level at baseline. This indicates that a universal intervention using MBSR as part of a teacher-training programme can reduce stress among lower secondary school teachers. Reducing the mean perceived stress level among school teachers with a moderate stress level may prevent these individuals from developing high stress levels.

## Supplementary data

Supplementary data are available at *EURPUB* online.

## Supplementary Material

ckab223_Supplementary_Data
