# Supplementary material for: The effectiveness of mindfulness-based stress reduction for school teachers: a cluster-randomized controlled trial
Source: Eur J Public Health. 2022 Feb 10;32(2):246–53. doi: 10.1093/eurpub/ckab223 (PMC8975540; doi:10.1093/eurpub/ckab223)
Supplement: ckab223_Supplementary_Data [file ckab223_supplementary_data.pdf]

Supplemental Online Content

Content and table-headings:

eTable 1: Loss to follow-up analysis at 6 months; included participants and participants lost to 6-months follow-up in both the MBSR intervention group and the waitlist control group

eTable 2: Loss to follow-up analysis at 6 months in the intervention group; included participants and participants lost to 6-months follow-up in the MBSR intervention group

eTable 3: Loss to follow-up analysis at 6 months in the wait-list control group; included participants and participants lost to 6-months follow-up in the wait-list control group

eTable 4: Sensitivity analysis of perceived stress level, symptoms of depression and anxiety and wellbeing

eTable 5: Sensitivity analysis of resilience, dispositional mindfulness and thoughts and feelings in rest

eTable 1: Loss to follow-up analysis at 6 months; included participants and participants lost to six months follow-up in both the MBSR intervention group and the waitlist control group.

Characteristic	Included	Lost to follow-up	p value
**Sex, n (%)**	n = 150	n = 41	
Men	13 (8.7)	3 (7.3)	0.782
Women	137 (91.3)	38 (92.7)	
**Age, mean (SD)**	46.0 (7.9)	42.3 (9.6)	0.0120
**Geographical region, n (%)**			
Central Denmark Region	41 (27.3)	9 (21.9)	0.015
The Capital Region of Denmark	51 (34.0)	5 (12.2)	
Region Zealand	21 (14.0)	7 (17.1)	
The Region of Southern Denmark	28 (18.7)	14 (34.2)	
The North Denmark Region	9 (6.0)	6 (14.6)	
**School type, n (%)**			
Private	47 (31.3)	15 (36.6)	0.524
Municipal	103 (68.7)	26 (63.4)	
**School size, (%)**			
≤499 pupils	76 (50.7)	24 (58.5)	0.371
≥500 pupils	74 (49.3)	17 (41.5)	
Self-reported mental health			
PSS, mean (SD)	15.9 (5.6)	15.8 (6.3)	0.923
SCL-5, mean (SD)	1.9 (0.5)	1.8 (0.6)	0.370
WHO-5, mean (SD)	59.0 (16.4)	59.6 (19.3)	0.849
BRS, mean (SD)	4.3 (0.9)	4.2 (0.7)	0.637
FFMQ-15, mean (SD)	41.9 (5.6)	42.1 (5.6)	0.875
ARSQ, mean (SD)			
Discontinuity of Mind	9.0 (2.7)	8.8 (2.5)	0.675
Theory of Mind	8.9 (2.8)	9.1 (2.7)	0.727
Self	9.6 (2.1)	8.8 (2.2)	0.0625
Planning	9.5 (2.9)	8.9 (2.9)	0.302
Sleepiness	6.4 (2.4)	7.0 (2.7)	0.136
Comfort	10.7 (2.0)	10.7 (2.0)	0.970
Somatic Awareness	10.7 (2.2)	9.9 (2.2)	0.0449

Test for no difference between included and missing participants at 6 months. Paired t-test was used for continuing variables, while Pearson's chi-squared test were used for categorical variables.

Abbreviations: ARSQ: Amsterdam Resting-State Questionnaire, BRS: Brief Resilience Scale, FFMQ: Five Facet Mindfulness Questionnaire, MBSR: Mindfulness-based Stress Reduction, n: number, PSS: Cohen's Perceived Stress Scale, SCL-5: The Hopkins Symptom Checklist 5, SD: standard deviation, WHO-5: WHO-5 Well-being Scale

eTable 2: Loss to follow-up analysis at 6 months in the intervention group; included participants and participants lost to six months follow-up in the MBSR intervention group

Characteristic	Included	Lost to follow-up	p value
Sex, n (%)	n = 77	n = 20	
Men	7 (9.1)	3 (15.0)	0.439
Women	70 (90.9)	17 (85.0)	
Age, mean (SD)	47.7 (7.9)	40.3 (9.4)	0.0005
Geographical region, n (%)			
Central Denmark Region	21 (27.3)	5 (25.0)	0.045
The Capital Region of Denmark	27 (35.0)	2 (10.0)	
Region Zealand	12 (15.6)	2 (10.0)	
The Region of Southern Denmark	13 (16.9)	8 (40.0)	
The North Denmark Region	4 (5.2)	3 (15.0)	
School type (%)			
Private	23 (29.9)	10 (50.0)	0.090
Municipal	54 (70.1)	10 (50.0)	
School size, (%)			
≤499 pupils	37 (48.1)	13 (65.0)	0.177
≥500 pupils	40 (51.9)	7 (35.0)	
Self-reported mental health			
PSS, mean (SD)	15.2 (5.3)	16.6 (6.2)	0.342
SCL-5, mean (SD)	1.9 (0.5)	1.8 (0.5)	0.513
WHO-5, mean (SD)	60.6 (16.5)	56.0 (18.5)	0.294
BRS, mean (SD)	4.4 (0.9)	4.2 (0.7)	0.500
FFMQ-15, mean (SD)	42.2 (5.6)	39.7 (4.8)	0.0585
ARSQ, mean (SD)			
Discontinuity of Mind	9.0 (2.7)	9.1 (2.3)	0.891
Theory of Mind	8.5 (2.8)	9.3 (2.5)	0.289
Self	9.3 (2.3)	8.4 (2.0)	0.136
Planning	9.0 (3.0)	9.2 (2.5)	0.817
Sleepiness	6.3 (2.5)	8.0 (2.8)	0.011
Comfort	10.7 (1.9)	10.8 (1.8)	0.909
Somatic Awareness	10.6 (2.2)	9.7 (2.4)	0.117

Abbreviations: ARSQ: Amsterdam Resting-State Questionnaire, BRS: Brief Resilience Scale, FFMQ: Five Facet Mindfulness Questionnaire, MBSR: Mindfulness-based Stress Reduction, n: number, PSS: Cohen's Perceived Stress Scale, SCL-5: The Hopkins Symptom Checklist 5, SD: standard deviation, WHO-5: WHO-5 Well-being Scale

eTable 3: Loss to follow-up analysis at 6 months in the wait-list control group; included participants and participants lost to six months follow-up in the wait-list control group

Characteristic	Included	Lost to follow-up	p value
Sex, n (%)	n = 73	n = 21	
Men	6 (8.2)	0 (0.0)	0.175
Women	67 (91.8)	21 (100.0)	
Age, mean (SD)	44.2 (7.7)	44.2 (9.6)	0.999
Geographical region, n (%)			
Central Denmark Region	20 (27.4)	4 (19.0)	0.250
The Capital Region of Denmark	24 (32.9)	3 (14.3)	
Region Zealand	9 (12.3)	5 (23.8)	
The Region of Southern Denmark	15 (20.5)	6 (28.6)	
The North Denmark Region	5 (6.9)	3 (14.3)	
School type (%)			
Private	24 (32.9)	5 (23.8)	0.428
Municipal	49 (67.1)	16 (76.2)	
School size, (%)			
≤499 pupils	39 (53.4)	11 (52.4)	0.933
≥500 pupils	34 (46.6)	10 (47.6)	
Self-reported mental health			
PSS, mean (SD)	16.6 (5.9)	15.1 (6.4)	0.316
SCL-5, mean (SD)	2.0 (0.6)	1.9 (0.7)	0.517
WHO-5, mean (SD)	57.3 (16.2)	63.0 (19.9)	0.191
BRS, mean (SD)	4.3 (0.9)	4.3 (0.7)	0.982
FFMQ-15, mean (SD)	41.4 (5.7)	44.4 (5.5)	0.0393
ARSQ, mean (SD)			
Discontinuity of Mind	9.1 (2.8)	8.6 (2.7)	0.481
Theory of Mind	9.3 (2.6)	8.9 (3.0)	0.528
Self	9.8 (1.9)	9.2 (2.4)	0.223
Planning	10.0 (2.7)	8.7 (3.3)	0.0831
Sleepiness	6.5 (2.3)	6.2 (2.3)	0.589
Comfort	10.6 (2.0)	10.6 (2.1)	0.884
Somatic Awareness	10.8 (2.3)	10.1 (2.1)	0.200

Abbreviations: ARSQ: Amsterdam Resting-State Questionnaire, BRS: Brief Resilience Scale, FFMQ: Five Facet Mindfulness Questionnaire, n: number, PSS: Cohen's Perceived Stress Scale, SCL-5: The Hopkins Symptom Checklist 5, SD: standard deviation, WHO-5: WHO-5 Well-being Scale

eTable 4: Sensitivity analysis of perceived stress level, symptoms of depression and anxiety and wellbeing

	MBSR intervention			Wait-list control		
	Between-group difference	95% CI	p-value	Between-group difference	95% CI	p-value
**Primary outcome**						
**+ 0.2 SD***						
**PSS**						
3 months	-1.6	-3.1; -0.1	0.032	-2.1	-3.6; -0.6	0.005
6 months	-2.1	-3.6; -0.6	0.006	-2.6	-4.1; -1.1	0.001
**- 0.2 SD***						
**PSS**						
3 months	-2.1	-3.6; -0.6	0.006	-1.6	-3.1; -0.1	0.038
6 months	-2.6	-4.1; -1.1	0.001	-2.1	-3.6; -0.6	0.006
**Secondary mental health outcome measures**						
**+ 0.2 SD***						
**SCL-5**						
3 months	-0.1	-0.3; 0.02	0.091	-0.2	-0.3; -0.03	0.020
6 months	-0.1	-0.2; 0.07	0.278	-0.1	-0.3; 0.02	0.087
**WHO-5**						
3 months	6.1	1.3; 10.8	0.013	4.5	-0.2; 9.3	0.062
6 months	5.0	0.2; 9.8	0.040	3.6	-1.2; 8.3	0.141
**- 0.2 SD***						
**SCL-5**						
3 months	-0.2	-0.3; -0.02	0.024	-0.1	-0.3: 0.03	0.105
6 months	-0.1	-0.3; 0.02	0.086	-0.08	-0.2; 0.07	0.276
**WHO-5**						
3 months	4.5	-0.2; 9.3	0.060	6.1	1.3; 10.9	0.013
6 months	3.5	-1.3; 8.2	0.150	4.9	0.1; 9.7	0.044

* Missing values were replaced by model-based predictions adding or subtracting 0.2 SD in either the intervention or the wait-list control arm. Thus, when adding or subtracting 0.2 SD to the intervention group, the wait-list control group estimate was kept constant. When adding or subtracting 0.2 SD to the wait-list control group, the intervention group estimate was kept constant.

Abbreviations: CI: confidence interval, MBSR: Mindfulness-based Stress Reduction, PSS: Cohen's Perceived Stress Scale, SCL-5: The Hopkins Symptom Checklist-5, SD: standard deviation, WHO-5: The WHO-5 Well-being Scale.

eTable 5: Sensitivity analysis of resilience, dispositional mindfulness and thoughts and feelings in rest						
	MBSR intervention			Wait-list control		
	Between-group difference	95% CI	p-value	Between-group difference	95% CI	p-value
**+ 0.2 SD***						
**BRS**						
3 months	0.2	-0.1; 0.4	0.067	0.1	-0.1; 0.3	0.309
6 months	0.07	-0.1; 0.3	0.473	-0.006	-0.2; 0.2	0.949
**FFMQ-15**						
3 months	2.1	0.9; 3.4	0.001	1.6	0.4; 2.8	0.011
6 months	1.7	0.4; 2.9	0.008	1.2	-0.06; 2.4	0.062
**ARSQ**						
Discontinuity of mind						
3 months	-1.3	-2.1; -0.6	0.001	-1.6	-2.3; -0.8	<0.001
6 months	-0.9	-1.6; -0.1	0.029	-1.1	-1.8; -0.3	0.006
Theory of mind						
3 months	-0.3	-1.1; 0.5	0.439	-0.6	-1.4; 0.3	0.183
6 months	-0.2	-1.0; 0.6	0.618	-0.4	-1.2; 0.4	0.297
Self						
3 months	-0.01	-0.7; 0.7	0.970	-0.2	-0.9; 0.5	0.538
6 months	0.7	0.03; 1.4	0.042	0.5	-0.2; 1.2	0.145
Planning						
3 months	-1.4	-2.2; -0.5	0.002	-1.6	-2.5; -0.8	<0.001
6 months	-0.4	-1.3; 0.4	0.341	-0.7	-1.5; 0.2	0.124
Sleepiness						
3 months	-1.0	-1.8; -0.2	0.016	-1.2	-2.1; -0.4	0.003
6 months	-0.9	-1.7; -0.05	0.038	-1.1	-1.9; -0.3	0.010
Comfort						
3 months	0.6	0.003; 1.2	0.049	0.4	-0.2; 1.0	0.167
6 months	0.9	0.3; 1.5	0.002	0.8	0.2; 1.4	0.013
Somatic awareness						
3 months	1.0	0.3; 1.7	0.004	0.8	0.1; 1.5	0.024
6 months	1.1	0.4; 1.7	0.003	0.9	0.2; 1.6	0.016
**- 0.2 SD***						
**BRS**						
3 months	0.1	-0.1; 0.3	0.275	0.2	-0.005; 0.4	0.056
6 months	-0.007	-0.2; 0.2	0.944	0.07	-0.1; 0.3	0.475
**FFMQ-15**						
3 months	1.6	0.4; 2.8	0.010	2.11	0.9; 3.3	0.001
6 months	1.2	-0.04; 2.4	0.058	1.7	0.5; 2.9	0.007
**ARSQ**						
Discontinuity of mind						
3 months	-1.5	-2.3; -0.8	<0.001	-1.3	-2.1; -0.5	0.001
6 months	-1.1	-1.9; -0.3	0.004	-0.9	-1.6; -0.1	0.023
Theory of mind						
3 months	-0.5	-1.3; 0.3	0.199	-0.3	-1.1; 0.5	0.469
6 months	-0.4	-1.3; 0.4	0.288	-0.2	-1.0; 0.6	0.604
Self						

3 months	-0.2	-0.9; 0.5	0.560	-0.001	-0.7; 0.7	0.998
6 months	0.5	-0.2; 1.2	0.155	0.7	0.01; 1.4	0.045
Planning						
3 months	-1.6	-2.4; 0.7	<0.001	-1.3	-2.2; -0.5	0.002
6 months	-0.7	-1.5; 0.2	0.117	-0.4	-1.3; 0.4	0.325
Sleepiness						
3 months	-1.2	-2.0; -0.4	0.005	-1.0	-1.8; -0.2	0.021
6 months	-1.1	-1.2; -0.3	0.010	-0.9	-1.7; -0.06	0.036
Comfort						
3 months	0.4	-0.2; 1.0	0.152	0.6	0.02; 1.2	0.044
6 months	0.8	0.2; 1.4	0.014	0.9	0.3; 1.5	0.002
Somatic awareness						
3 months	0.8	0.1; 1.5	0.020	1.1	0.4; 1.8	0.003
6 months	0.9	0.2; 1.6	0.016	1.1	0.4; 1.8	0.003

* Missing values were replaced by model-based predictions adding or subtracting 0.2 SD in either the intervention or the wait-list control arm. Thus, when adding or subtracting 0.2 SD to the intervention group, the wait-list control group estimate was kept constant. When adding or subtracting 0.2 SD to the wait-list control group, the intervention group estimate was kept constant.

Abbreviations: ARSQ: Amsterdam Resting-State Questionnaire, BRS: Brief Resilience Scale, FFMQ: Five Facet Mindfulness Questionnaire, CI: confidence interval, MBSR: Mindfulness-based Stress Reduction, SD: standard deviation.
